# Insights on *Anabaena* sp. PCC 7120 Responses to HCH Isomers: Tolerance, Degradation, and Dynamics on Potential *lin* Genes Expression

**DOI:** 10.1002/mbo3.70105

**Published:** 2025-12-02

**Authors:** Jorge Guío, María Luisa Peleato, Emma Sevilla

**Affiliations:** ^1^ Department of Biochemistry & Molecular and Cellular Biology University of Zaragoza Zaragoza Spain; ^2^ Institute for Biocomputation and Physics of Complex Systems (BIFI) Zaragoza Spain

**Keywords:** *Anabaena*, bioremediation, HCH isomers, *lin* genes

## Abstract

Hexachlorocyclohexane (HCH) was extensively used as a pesticide until the 1990s. It was synthesized by benzene photochlorination, resulting in a mixture of stereoisomers, which included α‐, β‐, γ‐, and δ‐HCH, among others. It was later discovered that only the γ‐HCH isomer (also called lindane) had insecticidal properties, so it began to be purified from this mixture, while the remaining HCH isomers (representing around 85%–90% of industrial HCH production) were disposed of in dumpsites, generating environmental issues. Several works have studied microbial‐driven biodegradation and physiological responses to γ‐HCH, but information concerning the other isomers is scarce. Since previous research showed that the cyanobacterium *Anabaena sp*. PCC 7120 is effective at removing lindane; this study focused on its responses to the α‐, β‐, and δ‐HCH isomers. The results showed that *Anabaena* tolerates α‐ and γ‐HCH well, with little impact on growth, while β‐ and δ‐HCH are more poorly tolerated and negatively affect growth and cell physiology. It was also found that, in the presence of *Anabaena* sp. PCC 7120, both α‐ and γ‐HCH are completely eliminated from supernatants while β‐ and δ‐HCH are partially eliminated. Additionally, the *linC* gene was found to be expressed at twice the normal level in the presence of α‐ and γ‐HCH at 2 mg/mL. Overall, this study reveals how *Anabaena* responds to key HCH isomers found in contaminated sites and supports its potential use in bioremediation.

## Graphical abstract

This study investigates the physiological responses of *Anabaena* sp. PCC 7120 to HCH isomers, emphasizing both its tolerance to these compounds and its ability to biodegrade them. The findings support the potential application of *Anabaena* in the bioremediation of environments contaminated with specific HCH isomers.

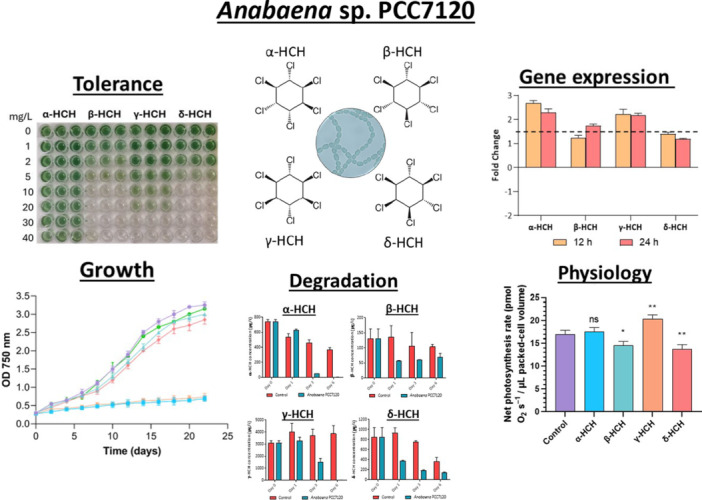

## 1 Introduction

Hexachlorocyclohexane (HCH) is an organochlorine compound that has been widely used as a pesticide in agriculture and as an insecticide for the control of vector‐borne diseases until the end of the 1990s (Li 1999). HCH is obtained by photochlorination of benzene, through a chain reaction in which Cl_2_ is homolysed by UV radiation, resulting in Cl· radicals that are added to the benzene molecule (Vijgen et al. 2006). This yields a mixture of stereoisomers known as technical lindane (t‐HCH), composed mainly of four HCH isomers: α‐HCH (60%–70%), β‐HCH (5%–12%), γ‐HCH (10%–15%), and δ‐HCH (6%–10%), and minimum amounts of the ϵ‐HCH, η‐HCH, and θ‐HCH (Vijgen et al. 2006).

Initially, HCH was applied as t‐HCH, but later on, it was proved that γ‐HCH, also referred to as lindane, was the only isomer in t‐HCH exhibiting insecticidal properties and was relatively more biodegradable (Slade 1945). For this reason, lindane began to be extracted and purified from t‐HCH, although t‐HCH was widely used as an insecticide in developing countries (Lal et al. 2010). Thus, lindane production involved that around 85%–90% of HCH generated in industrial production, which was commonly known as HCH muck, was discarded. In fact, it is estimated that, for each ton of lindane, between 8 and 12 tons of HCH muck were generated, which mainly consisted of α‐, β‐, and δ‐HCH isomers (Vijgen et al. 2006). Frequently, these residual isomers were improperly managed and were buried or disposed of in landfills from which the isomers can leach into the environment (Lal et al. 2010). This has generated heavily HCH‐contaminated sites all around the world, including Lucknow (India), Rio de Janeiro (Brazil), Sabiñánigo (Spain), and Pontevedra (Spain), among others (Nayyar et al. 2014).

HCH isomers have been described as human carcinogens and endocrine disruptors with carcinogenic, teratogenic, and neurological effects (Nayyar et al. 2014). This fact, together with its highly persistent and recalcitrant nature, led to its prohibition in many countries and its classification as a persistent organic pollutant (POP) by the Stockholm Convention in 2009 (Vijgen et al. 2011). In spite of the recent ban on the production and use of HCH, the extensive use of technical HCH and lindane over the years, together with the unregulated disposal of HCH industrial residues and their high persistence, has generated a serious concern for human health and an environmental problem in different parts of the world. This has raised interest in the development of bioremediation and biodegradation strategies to tackle this issue (Lal et al. 2010).

Although HCH is durable and recalcitrant, several bacterial strains able to degrade HCH have been identified (Lal et al. 2010). The first reports of microbial degradation of HCH isomers date back to the 1960s. First, anaerobic degradation of α‐HCH, β‐HCH, γ‐HCH, and δ‐HCH isomers was described in anaerobic microorganisms from the genera *Clostridium*, *Citrobacter*, *Desulfovibrio*, and *Desulfococcus* (Lal et al. 2010). It was observed that the γ‐HCH and α‐HCH isomers were degraded more rapidly than δ‐ and β‐HCH and that the degradation consisted basically of successive dichloroeliminations and dehydrochlorinations giving rise to products, such as 3,4,5,6‐tetrachloro‐1‐cyclohexene (3,4,5,6‐TCCH), chlorobenzene, and benzene (Lal et al. 2010).

Subsequently, aerobic degradation was discovered in members of the Sphingomonadaceae family isolated from HCH‐contaminated areas, being the most important ones *Sphingobium japonicum* UT26, *Sphingobium indicum* B90A, and *Sphingobium francense* Sp+ (Nagata et al. 2007). This aerobic route involves a set of genes called *lin* genes, which, unlike other genes involved in the degradation of pollutants, are not part of an operon, but are dispersed throughout the genome (Suar et al. 2004). Among all the HCH isomers, the one that can be degraded most easily is γ‐HCH. In *S. japonicum* UT26, the lindane degradation pathway is divided into an upper and a lower pathway. The upper pathway comprises *linA* (HCH dechlorinase), *linB* (haloalkane dehalogenase), and *linC* (dehydrogenase) and allows the transformation of lindane into 2,5‐dichlorohydroquinone (2,5‐DCHQ). Then, the downstream degradation pathway converts 2,5‐DCHQ into β‐ketoadipate thanks to the gene products of *linD* (reductive dechlorinase), *linE* (ring leavage oxygenase), and *linF* (maleylacetate reductase), which can be later transformed into succinyl‐CoA and acetyl‐CoA, which are metabolized by the Krebs cycle (Nagata et al. 1999).

However, as mentioned before, as γ‐HCH is removed during the purification process, the huge stockpiles of HCH muck consist mainly of α‐, β‐, and δ‐HCH isomers (Nayyar et al. 2014). Thus, α‐, β‐, and δ‐HCH degradation is more interesting from an environmental point of view. α‐HCH degradation has been studied in *S. indicum* B90A. This isomer can be transformed into β‐pentachlorocyclohexene by LinA and then spontaneously generate 1,2,4‐trichlorobenzene (1,2,4‐TCB), which is the dead‐end product (Lal et al. 2010). However, it has been suggested that, as it happens with the γ‐HCH, α‐HCH could also be mineralized to CO_2_ and H_2_O by a similar pathway (Suar et al. 2004; Lal et al. 2010), but the specific degradation route and intermediaries have not been described. The β‐HCH isomer is the most recalcitrant one, as it has all substituents in equatorial position, preventing dehydrochlorination reactions. In *S. indicum* B90A, it has been described that it can be converted into 2,3,5,6‐tetrachlorocyclohexanediol (2,3,5,6‐TCDL) via two successive hydrolytic dechlorination reactions catalyzed by LinB (Lal et al. 2010). With respect to δ‐HCH, it can be either partially degraded by the γ‐HCH route to generate 2,5‐DCHQ or be converted into 2,3,5,6‐TCDL via two hydrolytic dechlorinations, as it happens with β‐HCH (Lal et al. 2010). Interestingly, in *S. indicum* B90A, it has been described that the *linE* and *linD* genes are induced in the presence of 7 mg/L of γ‐HCH and 2 mg/L of α‐HCH by pathway intermediates through the action of LinR, which is a transcriptional activator, whereas the rest of the genes are constitutive (Suar et al. 2004).

Apart from heterotrophic bacteria, cyanobacteria have also proved to be good candidates for lindane degradation (Kuritz and Wolk 1995). The best studied one is *Anabaena* sp. PCC 7120, a nitrogen‐fixing cyanobacterium whose tolerance, physiological responses, and transcriptional profile of potential *lin* genes in response to lindane at its solubility limit in water have been analyzed (Guío et al. 2023). It was found that, in spite of inducing oxidative stress responses, lindane is well‐tolerated by this organism and can be degraded, yielding intermediates such as trichlorobenzene. This study also identified putative orthologs of some of the *lin* genes from *Sphingomonas paucimobilis* B90A, namely *linB, linC, linE,* and *linR*, and revealed that one of them, *linC*, was induced in the presence of lindane (Guío et al. 2023).

However, the tolerance and degradation of the other HCH isomers (α‐, β‐, and δ‐HCH) in cyanobacteria have not been explored. As these isomers pose important environmental and health problems and constitute the major HCH isomers present in polluted areas, we sought to investigate the ability of *Anabaena* sp. PCC 7120 to tolerate and degrade α, β, and δ‐HCH, as well as study its transcriptional and physiological responses in the presence of these compounds.

## 2 Materials and Methods

### 2.1 Strains and Culture Conditions

The axenic strain *Anabaena* sp. PCC 7120 was provided by the Pasteur Culture Collection (Paris, France) and was cultured in BG‐11 medium (Stanier et al. 1979) in which ammonium ferric citrate was replaced by FeSO_4_ to avoid inhibition of HCH metabolization (Kuritz et al. 1997). Cultures were maintained in a 250 mL Erlenmeyer flask containing 100 mL of cell culture at 28°C on a New Brunswick Innova 43 Shaker at 100 rpm under a continuous light regime of 30 μmol photons m^−2^ s^−1^ of white light. To analyze the responses of *Anabaena* sp. PCC 7120 to HCH isomers (α‐, β‐, γ‐, and δ‐HCH), 10 μL of solutions of 20 mg/mL of each HCH isomer in DMSO were added to 100 mL of cell culture, obtaining a final concentration of 2 mg/L.

### 2.2 Tolerance Assays

*Anabaena* sp. PCC 7120 tolerance to HCH isomers was measured in triplicate in 96‐well cell culture plates (CELLSTAR) using a method modified from Oyebamiji et al. (2021). *Anabaena* sp. PCC 7120 was grown to an OD_750 nm_ of 1.0, washed with fresh medium, and 200 μL of culture was dispensed into each well. Each HCH isomer (α‐, β‐, γ‐, and δ‐HCH) was tested separately and was added to the wells at a final concentration of 0, 1, 2, 5, 10, 20, 30, and 40 mg/L. The plate was incubated for 48 h at 28°C under a continuous light regime of 30 μmol photons m^−2^ s^−1^ of white light and gentle shaking at 100 rpm. Tolerance was evaluated by determining chlorosis, which was estimated by reading the absorbance at 620 nm with a Multiskan EX microplate photometer (Thermo Fisher Scientific).

### 2.3 Growth Curves

Photoautotrophic growth of *Anabaena* sp. PCC 7120 was measured in triplicate in the presence of 2 mg/L of each HCH isomer (α‐, β‐, γ‐, and δ‐HCH). Cultures were started with an initial OD_750 nm_ of 0.3, and untreated cells served as a control. Growth was monitored both by measuring the optical density and the packed cell volume (PCV) for 22 days every 48 h. Optical density was measured at 750 nm using a Cary 100 Bio UV‐Visible spectrophotometer (Varian). Packed cell volume was determined with 5 mL graduated tubes of 60 μL of packed volume capacity, and 5 mL of cell culture was added to the tubes and centrifuged for 5 min at 18°C and 2000 *g* in an Allegra X 30R centrifuge. PCV measurements were expressed as μL of packed volume per mL of cell culture.

### 2.4 Pigment Content Determinations

Pigment composition of *Anabaena* sp. PCC 7120 was determined after 48 h of exposure to 2 mg/L of each HCH isomer (α‐, β‐, γ‐, and δ‐HCH) in three independent cultures with an initial OD_750 nm_ of 0.3. Chl *a*, phycobiliproteins, and carotenoids levels were quantified as described by Mackinney (1941), Glazer (1976), and Davies (1976), respectively, and were normalized to packed‐cell volume.

### 2.5 Net Photosynthesis and Dark Respiration Rates Measurements

Net photosynthesis and dark respiration rates of *Anabaena* sp. PCC 7120 after 48 h of exposure to 2 mg/L of each HCH isomer (α‐, β‐, γ‐, and δ‐HCH) was measured at room temperature with a Clark‐type oxygen electrode model Chlorolab 2 (Hansatech). Net photosynthesis (considered as true photosynthesis minus photorespiration and dark respiration [Wohlfahrt and Gu 2015]) was determined by measuring oxygen release of 1 mL of cell culture illuminated with white light at 100 µmol photons m^−2^ s^−1^. Dark respiration was determined by measuring the oxygen consumption of 1 mL of cell culture in darkness. Both rates were measured in three independent replicates and were expressed as pmol O_2_·s^−1^/μL of packed‐cell volume.

### 2.6 RNA Extraction and Real‐Time RT‐PCR

Gene expression in the presence of HCH isomers was analyzed after 12 and 24 h of exposure to 2 mg/L of each HCH isomer (α‐, β‐, γ‐, and δ‐HCH) in three independent cultures with an initial OD_750 nm_ of 0.3. RNA was extracted and purified from 25 mL of culture as described previously (Sarasa‐Buisan et al. 2022). cDNA was synthesized by reverse transcription of 2 µg of total RNA using SuperScript retrotranscriptase (Invitrogen) following the manufacturer's instructions. Real‐time PCR was performed using the QuantStudio 5 Real‐Time PCR System (Applied Biosystems), and specific primers for each analyzed gene are included in Table S1. Each reaction was set up mixing 12.5 µL of SYBR Green PCR Master Mix with 0.4 μL of 25 µM primer mixture and 10 ng of cDNA template in a final volume of 30 µL. Amplification was performed at 60°C, and negative controls with no cDNA were included. Transcript levels of target genes were normalized to the housekeeping gene *rnpB* (Vioque 1992). Relative quantification was performed according to the comparative *C*_t_ method (ΔΔ*C*_t_ Method) (Livak and Schmittgen 2001), and the minimum fold‐change threshold was set up to ± twofold.

### 2.7 Degradation Measurements

Degradation of HCH isomers was measured in triplicate in 100 mL of *Anabaena* sp. PCC 7120 culture with an initial OD_750 nm_ of 0.6 containing 2 mg/L of each HCH isomer (α‐, β‐, γ‐, and δ‐HCH). Erlenmeyer flasks with 100 mL of BG‐11 medium without cells containing the same amount of HCH served as controls for evaporation and photodegradation. HCH degradation was measured after 1, 3, and 6 days of treatment, in supernatants after centrifugation of 10 mL of each sample for 5 min at 3000 *g*. HCH determinations were performed in the HCH Analysis laboratories at the facilities of the new security cell in Bailín II (Sabiñánigo, Huesca). Isomers of HCH in aqueous matrix were extracted using a liquid‐liquid extraction, and the quantification of the remaining amount of each isomer was carried out using gas chromatography with a mass detector (Agilent Series 7890A). The identification of isomers was carried out using commercial individual standards.

## 3 Results

### 3.1 *Anabaena sp*. PCC 7120 Shows Different Tolerance to HCH Isomers

**Figure 1:**
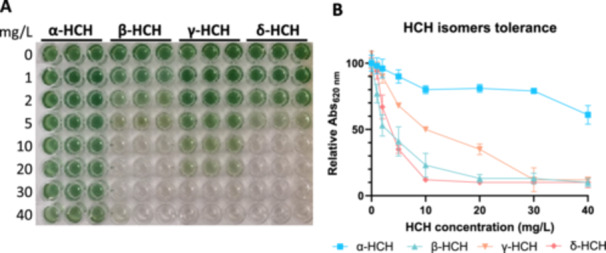
Tolerance of *Anabaena* sp. PCC 7120 to α‐, β‐, γ‐, and δ‐HCH isomers. (A) *Anabaena* sp. PCC 7120 was exposed in triplicate to increasing concentrations of α‐, β‐, γ‐, and δ‐HCH (0, 1, 2, 5, 10, 20, 30, and 40 mg/L) for 48 h, and results were documented by photography. The samples in the absence of HCH contained the same amount of DMSO as the rest of the wells to serve as a control for the effect of DMSO. (B) Estimation of chlorosis by reading wells' absorbance at 620 nm. The experiment was done twice with similar results.

To determine the tolerance of *Anabaena sp*. PCC 7120 to the four major HCH isomers, cells were exposed to increasing concentrations of α‐, β‐, γ‐, and δ‐HCH, namely 1, 2, 5, 10, 20, 30, and 40 mg/L for 48 h (Figure 1A,B). *Anabaena* sp. PCC 7120 showed a high tolerance to α‐HCH, since the relative Abs_620 nm_ was above 80% when exposed to 30 mg/L of α‐HCH, and it only decreased to 60% when exposed to 40 mg/L of this HCH isomer. The tolerance to γ‐HCH was moderate, as relative Abs_620 nm_ decreased to 50% when exposed to 10 mg/L, and concentrations above 30 mg/L produced practically total chlorosis. In the case of β‐HCH and δ‐HCH, the tolerance was low, as both isomers decreased relative Abs_620 nm_ to 50% at concentrations around 2 mg/L and produced practically total chlorosis above 10 mg/L. Consequently, all further studies were performed at 2 mg/L for the four isomers.

### 3.2 β‐HCH and δ‐HCH Negatively Affect the Growth of *Anabaena* sp. PCC 7120

**Figure 2:**
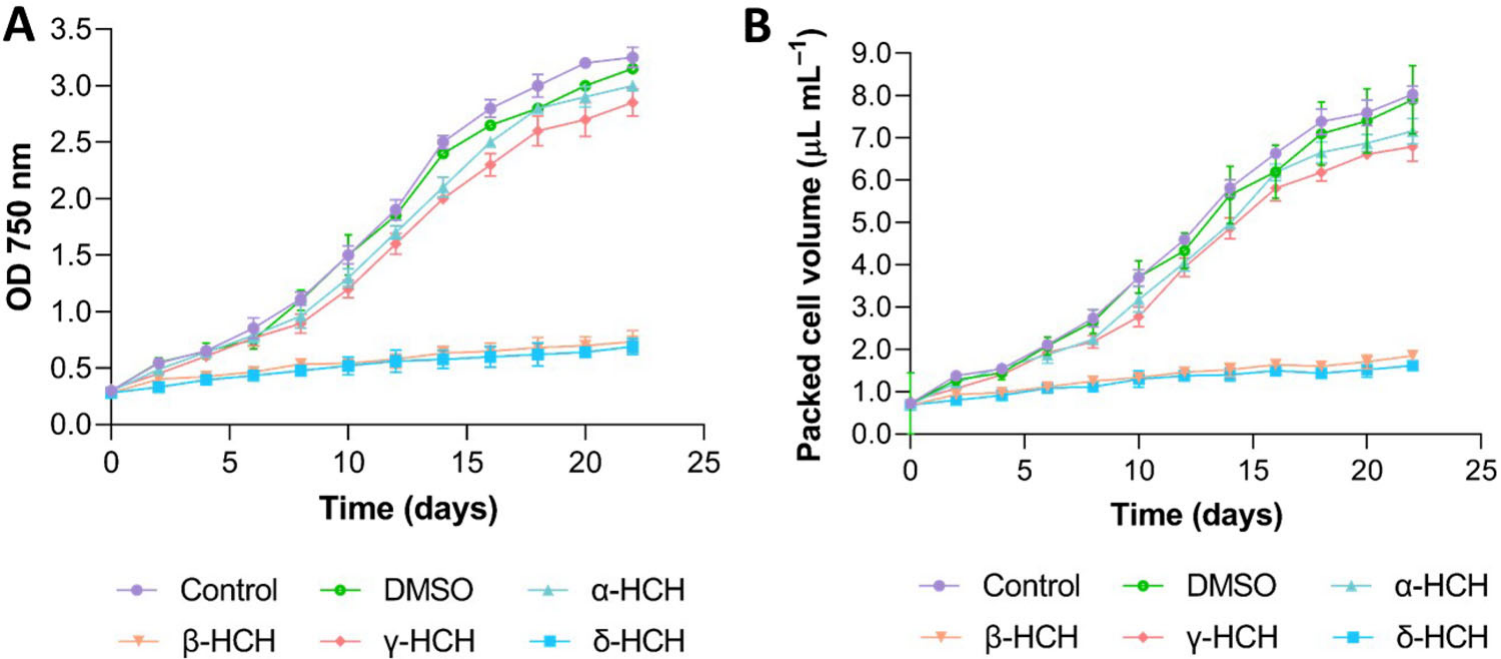
Growth curves of *Anabaena* sp. PCC 7120 in the presence of 2 mg/L of α‐, β‐, γ‐, or δ‐HCH for 22 days. (A) Optical densities at 750 nm of *Anabaena* sp. PCC 7120 cultures treated with 2 mg/L of α‐, β‐, γ‐, or δ‐HCH in comparison to untreated cells. The average of the three biological replicates is represented, and the standard deviation is included. (B) Packed cell volume of cultures treated with 2 mg/L of α‐, β‐, γ‐, or δ‐HCH. The average of the three biological replicates is represented, and the standard deviation is included. Control samples containing cells and DMSO in the absence of HCH are shown.

*Anabaena* cells' growth was monitored in triplicate in the presence of 2 mg/L of α‐, β‐, γ‐, or δ‐HCH for 22 days, and cells without HCH isomers served as a control. Previous works revealed that, at the concentration of work (10 μL in 100 mL of cell culture), DMSO used to dissolve HCH isomers had no effects on cell growth (Guío et al. 2023). Growth curves, obtained both by using OD_750 nm_ and packed‐cell volume (Figure 2A,B), revealed that the growth of *Anabaena* sp. PCC 7120 was similar to the untreated control in the presence of α‐ and γ‐HCH. In fact, the doubling time for untreated cells was 5.50 d^−1^, and doubling times in the presence of these two isomers were 5.87 and 5.96 d^−1^, respectively. However, cell growth was drastically affected by the presence of β‐HCH and δ‐HCH, which increased the doubling time to 19.70 and 19.66 d^−1^. Whereas cultures treated with α‐ and γ‐HCH reached an OD_750 nm_ of around 3.0 after 22 days, and the OD_750 nm_ in cells treated with β‐ and δ‐HCH was around 0.5 after 22 days of exposure. These results suggest that α‐ and γ‐HCH are well tolerated by *Anabaena* sp. PCC 7120, whereas β‐HCH and δ‐HCH seem to have detrimental effects on the growth of this cyanobacterium.

### 3.3 Effects of HCH Isomers on Pigment Composition, Photosynthetic Electronic Transport, and Respiration Rate

**Figure 3:**
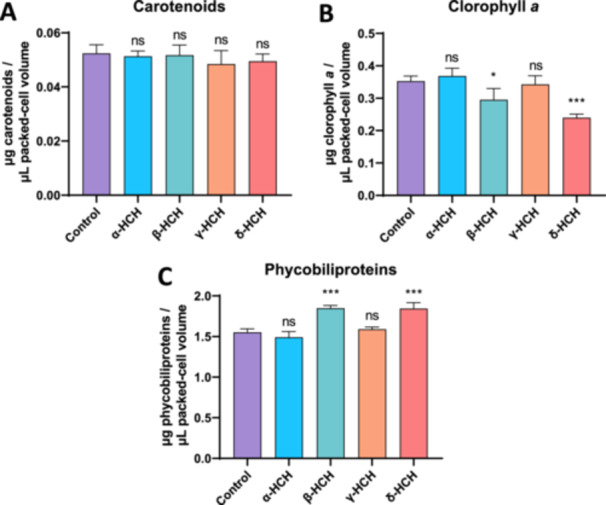
Pigment composition of *Anabaena* sp. PCC 7120 cells treated with 2 mg/L of α‐, β‐, γ‐, or δ‐HCH for 48 h. Cellular content of carotenoids (A), chlorophyll *a* (B), and phycobiliproteins (C) measured in three biological replicates of *Anabaena* sp. PCC 7120 cells after 48 h of exposure to 2 mg/L of α‐, β‐, γ‐, or δ‐HCH. Values were normalized to packed‐cell volume and expressed as μg of pigment μL^−1^ PCV. The average of the three measurements is represented, and the standard deviation is included. ns, no significant; *p < 0.05; ***p < 0.001, obtained with a *t*‐test analysis comparing each data with respect to untreated cells (control).

**Figure 4:**
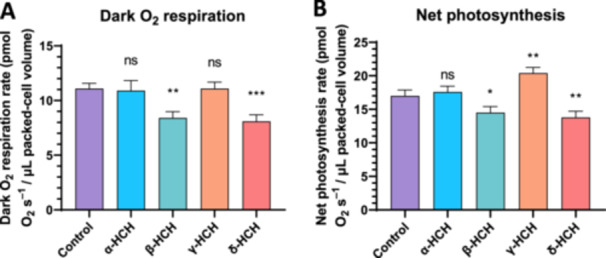
Net photosynthesis and dark respiration rates of *Anabaena* sp. PCC 7120 cells treated with 2 mg/L of α‐, β‐, γ‐, or δ‐HCH for 48 h. Net photosynthesis rate at 100 μmol photons m^–2^ s^–1^ (A) and dark respiration rate (B) measured in three biological replicates of *Anabaena* sp. PCC 7120 cells after 48 h of exposure to 2 mg/L of α‐, β‐, γ‐, or δ‐HCH. Values were normalized to packed‐cell volume and expressed as pmol O_2_ s^−1^ μL^−1^ PCV. The average of the three measurements is represented, and the standard deviation is included. ns, no significant; *p < 0.05; **p < 0.01, ***p < 0.001, obtained with a *t*‐test analysis comparing each data with respect to untreated cells (control).

To better understand the physiological effects of HCH isomers on *Anabaena* sp. PCC 7120, cellular contents of chlorophyll a, phycobiliproteins, and carotenoids (Figure 3), as well as photosynthesis and dark respiration rates (Figure 4), were measured after 48 h of exposure to 2 mg/L of α‐, β‐, γ‐, or δ‐HCH.

Figure 3A shows that the levels of carotenoids were not affected by the presence of any of the HCH isomers tested. However, as can be seen in Figure 3B,C, the levels of chlorophyll *a* and phycobiliproteins were significantly affected in cells treated with β‐HCH and δ‐HCH, whereas no changes were observed in the case of α‐HCH and γ‐HCH. In particular, β‐HCH and δ‐HCH produce a slight decrease in the levels of chlorophyll *a* (Figure 3B), whereas the levels of phycobiliproteins are moderately increased (Figure 3C).

With respect to photosynthesis and respiration rates, results revealed that β‐ and δ‐HCH decrease both photosynthesis and respiration rates with respect to untreated cells (Figure 4). The presence of α‐HCH did not affect either photosynthesis or respiration rates, whereas γ‐HCH produced a slight increase in the photosynthetic rate. This is in agreement with previous works (Bueno et al. 2004; Guío et al. 2023), which revealed that at its solubility limit in water (7 mg/L), lindane also increases photosynthesis rate.

Taken together, these results reveal that, in the presence of α‐HCH and γ‐HCH, the physiological status of *Anabaena* sp. PCC 7120 is well‐preserved, whereas β‐HCH and δ‐HCH seem to have negative effects on the physiology of this organism.

### 3.4 α‐HCH and γ‐HCH Trigger Oxidative Stress Responses in *Anabaena* sp. PCC 7120

**Figure 5:**
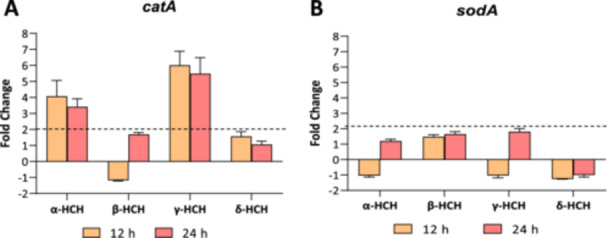
Relative transcription of genes involved in oxidative stress response in *Anabaena* sp. PCC 7120 cells treated with 2 mg/L of α‐, β‐, γ‐, or δ‐HCH. The relative transcription of superoxide dismutase A (*sodA*) (A) and catalase (*cat*) (B) was analyzed by Real‐Time RT‐PCR in *Anabaena* sp. PCC 7120 cells after 12 and 24 h of exposure to 2 mg/L of α‐, β‐, γ‐, or δ‐HCH with respect to untreated cells. Values are expressed as fold change (treated vs. control) and correspond to the average of three biological and three technical replicates. The standard deviation is indicated.

Previous works revealed that lindane induced oxidative stress responses in *Anabaena* sp. PCC 7120 at 7 mg/L (Guío et al. 2023). In fact, it triggered a strong upregulation in the expression of catalase (*cat*), whose transcription increased around 40‐fold after 24 h of exposure to 7 mg/L of lindane. In this study, the expression of superoxide dismutase A (*sodA*) and catalase (*cat*), two enzymes involved in the detoxification of ROS, was analyzed after 12 and 24 h of treatment with 2 mg/L of α‐, β‐, γ‐, and δ‐HCH. Figure 5 shows that, whereas β‐HCH and δ‐HCH do not produce changes in the expression levels of *cat* at the concentrations studied, both α‐HCH and γ‐HCH induce the expression of *cat.* Specifically, the expression is upregulated around fourfold after 12 h of exposure to 2 mg/L of α‐HCH and sixfold after 12 h of exposure to 2 mg/L of γ‐HCH, and it decreases slightly after 24 h of exposure for both isomers. The expression of *sodA* was not affected by the presence of any of the HCH isomers at the concentrations studied. These results suggest that, apart from lindane, α‐HCH also triggers oxidative stress responses in *Anabaena* sp. PCC 7120. In the case of β‐HCH and δ‐HCH, in spite of being more poorly tolerated, they seem not to induce oxidative stress responses under the studied conditions.

### 3.5 Degradation of HCH Isomers by *Anabaena* sp. PCC 7120

**Figure 6:**
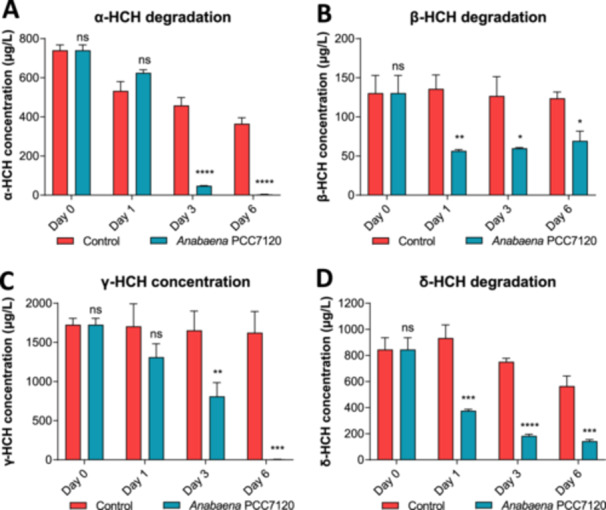
Analysis of HCH degradation by *Anabaena* sp. PCC 7120. HCH concentration in the supernatant of *Anabaena* sp. PCC 7120 cultures containing 2 mg/L of α‐HCH (A), β‐HCH (B), γ‐HCH (C), and δ‐HCH (D) after 1, 3, and 6 days of exposure. Samples containing BG‐11 medium without cells and the same amount of HCH served as controls for evaporation and photodegradation. ns, no significant; *p < 0.05; **p < 0.01; ***p < 0.001; ****p < 0.0001, obtained with a *t*‐test analysis comparing each data point with respect to its control.

*Anabaena* sp. PCC 7120 proved to be able to metabolize lindane almost totally after 6 days of exposure to 7 mg/L of this compound (Guío et al. 2023). To assess its ability to degrade other HCH isomers, *Anabaena* sp. PCC 7120 cells were incubated with 2 mg/L of either α‐, β‐, γ‐, or δ‐HCH in triplicate for 6 days. Degradation of each of the HCH isomers was determined by measuring HCH content in the supernatant of cultures after 1, 3, and 6 days. Results are shown in Figure 6.

In agreement with results obtained in previous studies (Guío et al. 2023), *Anabaena* was able to fully degrade 2 mg/L of γ‐HCH after 6 days of exposure. Interestingly, this cyanobacterium was also able to fully degrade 2 mg/L of α‐HCH after 6 days. Curiously, the degradation rate seems to be faster than for γ‐HCH, since α‐HCH concentration after 3 days was 6% of the initial one, whereas, in the case of lindane, after 3 days, the concentration only decreased to 47% of the initial one.

In the case of β‐HCH and δ‐HCH isomers, *Anabaena* proved to be able to partially degrade them. In the case of β‐HCH, the concentration in the presence of cells decreased to 45% after 6 days of exposure, and for the δ‐HCH isomer, the concentration was lowered to 20% of the initial one after 6 days in cell cultures.

### 3.6 Dynamics of Expression of Putative *lin* Genes in Response to HCH Isomers

**Figure 7:**
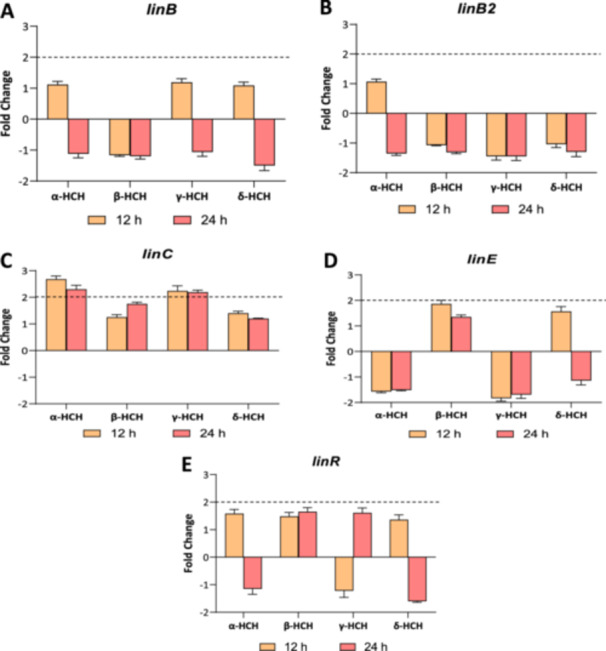
Relative transcription of putative *lin* genes in *Anabaena* sp. PCC 7120 cells treated with 2 mg/L of α‐, β‐, γ‐, or δ‐HCH. The relative transcription of putative *lin* genes *linB1* (A), *linB2* (B), *linC* (C), *linE* (D), and *linR* (E) was analyzed by real‐time RT‐PCR in *Anabaena* sp. PCC 7120 cells after 12 and 24 h of exposure to 2 mg/L of α‐, β‐, γ‐, or δ‐HCH with respect to untreated cells. Values are expressed as fold change (treated vs. control) and correspond to the average of three biological and three technical replicates. The standard deviation is indicated.

It has been suggested that *Anabaena* sp. PCC 7120 contains putative *lin* genes in its genome, namely, *linB1*, *linB2*, *linC, linE,* and *linR,* and it has been reported that the putative *linC* gene is induced 16‐fold after 12 h of exposure to 7 mg/L of lindane (Guío et al. 2023). In this study, the expression of the putative *lin* genes of *Anabaena* sp. PCC 7120 was analyzed after 12 and 24 h of exposure to 2 mg/L of α‐, β‐, γ‐, and δ‐HCH. As it can be seen in Figure 7, the transcription of *linB*, *linB2*, *linR,* and *linE* does not show significant changes in the presence of any of the isomer conditions tested. However, the expression of the *linC* gene shows a slight induction after 12 and 24 h of exposure to 2 mg/L of α‐HCH and to 2 mg/L γ‐HCH.

## 4 Discussion

As previously mentioned, as a consequence of the lindane manufacturing process, the α‐, β‐, and δ‐HCH isomers are the major HCH isomers present in many contaminated sites (Nayyar et al. 2014). Besides, HCH dumpsites frequently contain solvents employed in lindane extraction, which can increase the solubilization and mobilization of these isomers, posing an environmental threat. Cyanobacteria, in particular *Anabaena* sp. PCC 7120, are good candidate for HCH bioremediation. *Anabaena* sp. PCC 7120 has already been proven suitable for lindane bioremediation, since it was found that this cyanobacterium could tolerate the γ‐HCH isomer at its solubility limit in water (7 mg/L) and that this compound barely affected its physiological status (Guío et al. 2023). Besides, *Anabaena* also exhibited lindane degradation capacity, since γ‐HCH disappeared completely from supernatants of cells exposed to 7 mg/L of this compound for 6 days, and some degradation intermediaries were detected inside cells (Guío et al. 2023). However, to develop HCH bioremediation strategies based on *Anabaena* sp. PCC 7120, it is necessary to understand the effects of the other HCH isomers on this cyanobacterium.

In this study, we have analyzed the tolerance of *Anabaena* sp. PCC 7120 to the four major HCH isomers, namely α‐, β‐, γ‐, and δ‐HCH. We have found that γ‐HCH and especially α‐HCH are well tolerated by this organism. In the case of γ‐HCH, relative Abs_620 nm_ was above 50% for concentrations up to 10 mg/L, and in the case of α‐HCH, relative Abs_620 nm_ was above 80% for concentrations up to 30 mg/L. In both cases, these concentrations are widely superior to the solubility limits in water, which are considered to be 2 mg/L for α‐HCH and 7 mg/L for γ‐HCH (Nayyar et al. 2014), although it has been reported that in contaminated sites the solubility limit for α‐HCH could go up to 70 mg/L (Clayton et al. 1991) and for γ‐HCH up to 17 mg/L (Hollifield 1979) due to the presence of organic solvents that increase their solubility.

On the contrary, the tolerance to the β‐ and δ‐HCH isomers is low, since both isomers decrease relative Abs_620 nm_ to 50% at 2 mg/L and produce practically total chlorosis at 10 mg/L. However, as these isomers were generated in low percentages during the HCH industrial synthesis process, the concentrations found in contaminated places are usually low. For instance, measurements of HCH isomers in groundwaters at INQUINOSA production site in Sabiñanigo (Spain) determined that the concentrations of β‐HCH and δ‐HCH in water were 0.7 and 2.2 mg/L, respectively (Fernández et al. 2013). Consequently, although β‐HCH and δ‐HCH are more poorly tolerated, their low concentration in contaminated places will possibly allow the use of *Anabaena* for HCH bioremediation purposes in field conditions. However, before implementing bioremediation approaches based on the use of *Anabaena* sp. PCC 7120, it would be necessary to carefully study the levels of β‐HCH and δ‐HCH in contaminated places so as to avoid cell death because of these compounds.

Analysis of cell growth in the presence of HCH isomers revealed that α‐HCH and γ‐HCH do not significantly affect the growth rate at 2 mg/L, suggesting that a suitable physiological status of *Anabaena* is maintained in the presence of these compounds. On the contrary, β‐HCH and δ‐HCH at 2 mg/L, albeit not producing cell death, cause a drastic diminution in the growth rate of *Anabaena*, suggesting detrimental effects on cell physiology. The measurement of pigment composition, photosynthesis, and respiration rates of *Anabaena* sp. PCC 7120 after 48 h of exposure to these isomers is in agreement with cell growth data. In the case of α‐ and γ‐HCH, no significant changes are observed in pigment composition or in photosynthesis and respiration rates with respect to untreated cells, except for a slight increase in photosynthesis rate in the presence of lindane. This increment of the photosynthesis rate in the presence of lindane was reported previously (Bueno et al. 2004; Guío et al. 2023), and it has been proposed that it could be explained by the release of oxygen by other processes, such as the oxidative stress response via catalase (Guío et al. 2023).

On the contrary, both β‐ and δ‐HCH lower photosynthesis and respiration rates, revealing a significant effect of physiological status in the presence of these compounds. This diminution of the photosynthesis rate could account for the drastic reduction of the growth rate in the presence of these two isomers, whereas the decrease of the respiration rate could be a consequence of less energy being required due to the lower growth rate. Affectation of photosynthesis in the presence of HCH was previously reported in the presence of high concentrations of γ‐HCH, and it was attributed to a non‐competitive inhibition of PS II (Bueno et al. 2004; Suresh Babu et al. 2001). Consequently, β‐ and δ‐HCH might also act as a non‐competitive inhibitor of PS II at lower concentrations than γ‐HCH, explaining the decrease in photosynthesis rate and chlorophyll *a* levels. Besides, it was found that in the presence of β‐ and δ‐HCH, phycobiliprotein levels increase, which could be an adaptive response of cyanobacteria to increase light harvesting in photosystem II to compensate for the reduction in photosynthesis rate.

These results are in agreement with previous works with other organisms. For instance, Sharma et al. (2021) reported that, for *S. paucimobilis,* B90A growth was similar to the untreated control in the presence of α‐ and γ‐HCH, while it decreased significantly in the presence of δ‐ and β‐HCH. These differences in growth rate were attributed to the differential ability of strain B90A to degrade individual HCH isomers, as it was found that α‐ and γ‐HCH were completely degraded, whereas β‐HCH and δ‐HCH were only partially depleted (Sharma et al. 2021). Similar results were observed for *Bacillus* spp., which was able to almost fully degrade γ‐HCH, whereas β‐HCH was hardly degradable (Pannu and Kumar 2023). In the case of *Anabaena*, similar results have been obtained. Here, we report that α‐ and γ‐HCH are completely depleted in the presence of *Anabaena* sp. PCC 7120, while only 55% of β‐HCH and 80% of δ‐HCH are depleted from cultures exposed to these compounds for 6 days. This correlates with the growth rates of *Anabaena* in the presence of these compounds, which are barely affected by α‐ and γ‐HCH and are significantly reduced in the presence of β‐ and δ‐HCH.

Analysis of the degradation pathways in *Sphingomonas* revealed that γ‐HCH (and presumably α‐HCH) are completely mineralized, whereas β‐ and δ‐ are degraded only to chlorinated intermediaries, which could be toxic, causing reduced growth of strain B90A (Lal et al. 2010; Suar et al. 2004). Similar degradation pathways could be taking place in *Anabaena*, since both α‐ and γ‐HCH are well tolerated and fully depleted from the supernatant (although not totally mineralized, at least in the case of γ‐HCH (Guío et al. 2023)), whereas β‐ and δ‐HCH are partially depleted and have negative effects on the physiology of this cyanobacterium. Consequently, it could be the case that in *Anabaena* β‐ and δ‐HCH are also degraded to chlorinated intermediaries, which cannot be degraded further and produce a reduction of cell growth and negative effects on its physiology. Regarding environmental deployment, since *Anabaena* seems to be able to remove γ‐ and α‐HCH, transforming them into other compounds, such as trichlorobenzene (Guío et al. 2023), this cyanobacterium could be used in bioremediation processes combined, for example, with heterotrophic bacteria, with the aim of removing lindane from environmentally polluted water. It has been reported that the consortia between aerobic bacteria and cyanobacteria/microalgae can be efficient in the removal of organic pollutants from wastewaters, compared to the individual microorganisms (Subashchandrabose et al. 2011). For example, an artificial consortia between microalgae *Scenedesmus* sp. ISTGA1 and the bacterial strain *Paenibacillus* were used efficiently for the biodegradation of lindane (Kumari et al. 2020).

Previous studies revealed that the expression of the putative *linC* gene was induced around 16‐fold after 12 h of exposure to lindane (Guío et al. 2023). Here, it has been found that this induction, albeit not being so strong, also takes place at lower concentrations of lindane, since the exposure of 2 mg/L of γ‐HCH gives rise to a twofold increase in the expression of this gene both at 12 and 24 h. Interestingly, it has also been found that the expression of *linC* is also induced in the presence of 2 mg/L α‐HCH but not in the presence of β‐ or δ‐HCH. Thus, apparently, the induction of *linC* expression seems to take place exclusively in the presence of α‐ and γ‐HCH. In the present study, the induction of *linC* observed in the presence of lindane is lower than that reported in the previous article. This may be due to the fact that, here, a concentration of 2 mg/L was used (to standardize the concentrations of all isomers), and this is a lower concentration than the 7 mg/L used in the previous article. However, regarding the α‐HCH, it cannot be ruled out that the transcriptional response may vary in intensity depending on the isomer.

Similar results have been observed in other organisms, such as *S. paucimobilis* B90A. In this organism, as opposed to *Anabaena* sp. PCC 7120, *linC* is expressed constitutively, and *linD* and *linE* are induced in a process presumably controlled by the transcriptional regulator LinR (Suar et al. 2004). However, similarly to what happens in *Anabaena* sp. PCC 7120 with *linC*, in *S. paucimobilis*, B90A *linD* and *linE* are induced in the presence of α‐ and γ‐HCH but not in the presence of β‐ and δ‐HCH despite the fact that 50% of the compounds were degraded (Suar et al. 2004). Taken together, all these findings suggest that, as it happens in *Sphingomonas*, degradation of β‐ and δ‐HCH in *Anabaena* proceeds in a different pathway than that of α‐ and γ‐HCH. Consequently, it would be of interest to identify degradation intermediaries of α‐, β‐, γ‐, and δ so as to unveil the degradation pathways of these isomers in *Anabaena* sp. PCC 7120.

Finally, analysis of the expression of genes involved in oxidative stress response, namely *cat* and *sodA*, revealed that the transcription of *cat* is induced in the presence of α‐HCH and γ‐HCH, whereas its expression is not affected by either β‐HCH or δ‐HCH. The upregulation of catalase in the presence of 7 mg/L of lindane was already reported in previous works (Guío et al. 2023). Here, it was found that lower concentrations of this isomer (2 mg/L) are also able to induce the expression of this gene. Interestingly, it was also found that not only γ‐HCH, but also α‐HCH, induces the expression of *cat*, suggesting that both HCH isomers trigger oxidative stress responses in *Anabaena*. The fact that this induction was not observed in the presence of β‐ and δ‐HCH could be due to the fact that the oxidative stress response in the presence of these isomers happens at a different time frame than in the case of α‐ and γ‐HCH or that it is orchestrated by other mechanisms. However, another plausible explanation could be that this oxidative stress response is not triggered by HCH, but by intermediaries of degradation that are only generated by α‐ and γ‐HCH. Considering that both α‐ and γ‐HCH are well tolerated, both of them can be fully degraded from the supernatant, and the expression of *linC* is only induced in the presence of these two isomers, it could be the case that both isomers share degradation pathways and perhaps degradation intermediaries responsible for this oxidative stress response.

In conclusion, this study sheds new light on the tolerance and physiological responses of *Anabaena* sp. PCC 7120 to α‐, β‐, and δ‐HCH isomers, which are predominant in dumping sites, providing valuable information for the use of this organism in bioremediation approaches.

This study revealed that *Anabaena* sp. PCC 7120, which was previously reported as an appropriate organism for lindane bioremediation (Guío et al. 2023), also exhibits the ability to degrade α‐, β‐, and δ‐HCH isomers, even though β‐ and δ‐HCH cause detrimental effects on cells and degradation is less efficient. Besides, *linC* induction in the presence of α‐HCH and γ‐HCH isomers opens the door for the development of a whole‐cell biosensor for the detection of both HCH isomers.

## Author Contributions

**Jorge Guío:** conceptualization, investigation, data curation, writing – original draft, writing – review and editing. **María Luisa Peleato:** conceptualization, supervision, writing – original draft, writing – review and editing. **Emma Sevilla:** conceptualization, supervision, funding acquisition, project administration, writing – original draft, writing – review and editing.

## Ethics Statement

The authors have nothing to report.

## Conflicts of Interest

The authors declare no conflicts of interest.

## Supporting information


**Table S1:** Oligonucleotides used in this study.

## Data Availability

Data sharing is not applicable to this article as no new data were created or analyzed in this study.
