# Supplementary material for: Insights on Anabaena sp. PCC 7120 Responses to HCH Isomers: Tolerance, Degradation, and Dynamics on Potential lin Genes Expression
Source: Microbiologyopen. 2025 Dec 2;14(6):e70105. doi: 10.1002/mbo3.70105 (PMC12670186; doi:10.1002/mbo3.70105)
Supplement: Supplementary file 1 — Table S1: Oligonucleotides used in this study. [file MBO3-14-e70105-s001.docx]

| **Table S1.** Oligonucleotides used in this study | | |
| --- | --- | --- |
| Primer | Sequence (5’-3’) | Gene |
| *rnpB* for | AGCGGAACTGGTAAAAGACCAA | Gene *rnpB* from *Anabaena* sp. PCC 7120 |
| *rnpB* rev | GAGAGGTACTGGCTCGGTAAACC |  |
| *linB1* for | CAGCTTTGGCGGCTCAAG | Gene *all1353* from *Anabaena* sp. PCC 7120 |
| *linB1* rev | GAAAAGCCAGAACCAATCCAAT |  |
| *linB2* for | CGATCGCACTCTCAAAGCTATAATC | Gene *all0193* from *Anabaena* sp. PCC 7120 |
| *linB2* rev | TCACATAGTAGCGCCAGATATGC |  |
| *linC* for | GGAAAAGAAAGCAGTTGTAGAAAGTCA | Gene *all3836* from *Anabaena* sp. PCC 7120 |
| *linC* rev | GCTACTGCTGCCGCCATTT |  |
| *linE* for | ACTTCCGCATCTCTGCAAAAA | Gene *all0352* from *Anabaena* sp. PCC 7120 |
| *linE* rev | CAGCCGTCTATTTGGCAACA |  |
| *linR* for | AAATTCATGTTGCGTTGGGTTT | Gene *alr0353* from *Anabaena* sp. PCC 7120 |
| *linR* rev | CCAACAAAGTGTTCTTCAAATAATGG |  |
| *sodA* for | CTAACCAAACCCAACCACTACCA | Gene *sodA* from *Anabaena* sp. PCC 7120 |
| *sodA* rev | CCTTTGGCAGTTTTGAAGAGTTC |  |
| *catA* for | ACCCACGGGATGTCATTATGA | Gene *catA* from *Anabaena* sp. PCC 7120 |
| *catA* rev | TGGCAACGCACCTAAACCA |  |
